# Cardiac Resynchronization Therapy Delivered Via a Multipolar Left Ventricular Lead is Associated with Reduced Mortality and Elimination of Phrenic Nerve Stimulation: Long‐Term Follow‐Up from a Multicenter Registry

**DOI:** 10.1111/jce.12625

**Published:** 2015-03-05

**Authors:** JONATHAN M. BEHAR, JULIAN BOSTOCK, ADRIAN PO ZHU LI, HUI MEN SELINA CHIN, STEPHEN JUBB, EDWARD LENT, JAMES GAMBLE, PAUL W.X. FOLEY, TIM R. BETTS, CHRISTOPHER ALDO RINALDI, NEIL HERRING

**Affiliations:** ^1^ Department of Imaging Sciences and Bioengineering, BHF Centre of Research ExcellenceThe Rayne InstituteLondonUK; ^2^ Department of CardiologyGuy's and St Thomas's NHS Foundation TrustLondonUK; ^3^ Oxford Heart Centre, John Radcliffe HospitalOxford University Hospitals NHS TrustUK; ^4^ Great Western HospitalSwindonUK

**Keywords:** cardiac resynchronization therapy, implantable cardioverter defibrillator, left ventricular pacing, phrenic nerve stimulation, quadripolar lead

## Abstract

**Introduction:**

Cardiac resynchronization therapy (CRT) using quadripolar left ventricular (LV) leads provides more pacing vectors compared to bipolar leads. This may avoid phrenic nerve stimulation (PNS) and allow optimal lead placement to maximize biventricular pacing. However, a long‐term improvement in patient outcome has yet to be demonstrated.

**Methods:**

A total of 721 consecutive patients with conventional CRTD criteria implanted with quadripolar (n = 357) or bipolar (n = 364) LV leads were enrolled into a registry at 3 UK centers. Lead performance and mortality was analyzed over a 5‐year period.

**Results:**

Patients receiving a quadripolar lead were of similar age and sex to those receiving a bipolar lead, although a lower proportion had ischemic heart disease (62.6% vs. 54.1%, P = 0.02). Both groups had similar rates of procedural success, although lead threshold, impedance, and procedural radiation dose were significantly lower in those receiving a quadripolar lead. PNS was more common in those with quadripolar leads (16.0% vs. 11.6%, P = 0.08), but was eliminated by switching pacing vector in all cases compared with 60% in the bipolar group (P < 0.001). Furthermore, LV lead displacement (1.7% vs. 4.6%, P = 0.03) and repositioning (2.0% vs. 5.2%, P = 0.03) occurred significantly less often in those with a quadripolar lead. All‐cause mortality was also significantly lower in the quadripolar compared to bipolar lead group in univariate and multivariate analysis (13.2% vs. 22.5%, P < 0.001).

**Conclusions:**

In a large, multicenter experience, the use of quadripolar LV leads for CRT was associated with elimination of PNS and lower overall mortality. This has important implications for LV pacing lead choice.

## Introduction

The morbidity and mortality associated with heart failure remains high with 20–30% mortality at 3 years.^1^ Cardiac resynchronization therapy (CRT) is an effective treatment for patients with severe left ventricular dysfunction and broad left bundle branch block (LBBB).^2^, ^3^ Despite its complexity, CRT is a routine procedure, and due to advances in catheters, leads and increasing clinical experience, procedural success is now high. Major issues that limit the continuous effective delivery of this therapy are high pacing thresholds and phrenic nerve stimulation (PNS).^4^ The introduction of new multipolar (quadripolar) left ventricular leads (initially the Quartet 1458Q (St. Jude Medical, Sylmar, CA, USA) and more recently the Attain Performa (Medtronic Inc, Minneapolis, MN, USA) and Acuity X4 (Boston Scientific, Marlborough, Massachusetts, USA) allow greater programmability with 10–17 potential vectors.^5^, ^6^, ^7^ These leads give the implanting physician more choice compared with traditional bipolar LV leads and may also allow more distal lead placement with improved stability. This may reduce the need for reintervention in the lifespan of the device with the ability to program around most technical issues such as high thresholds and PNS post implantation.

PNS may occur at the site of optimal LV lead placement during implantation and is present in up to one‐fifth of patients.^8^ Although reprogramming bipolar leads may resolve this in some patients, there is often a need for another procedure to revise the LV lead position. In some circumstances, persistent and refractory PNS results in the LV lead being turned off, negating the overall benefit of CRT delivery. Since the introduction of quadripolar leads over 5 years ago, several small studies have reported higher implant success rates, lower rates of lead displacement and low rates of PNS both acutely and during 6 months of follow‐up.^9^, ^10^, ^11^ However, longer term follow‐up looking at PNS and mortality data has not been reported. We report the experience of 3 UK centers over 5 years with quadripolar leads, as compared with traditional bipolar LV leads in delivering CRT.

## Patients and Methods

A total of 1,104 patients with conventional CRT indications were implanted at 3 UK hospitals (Guy's and St Thomas’ Hospital NHS Foundation Trust, London [n = 599], John Radcliffe Hospital, Oxford [n = 397], and The Great Western Hospital, Swindon [n = 108]), between January 2009 and January 2014. All patients provided fully informed consent. Both *de novo* implantation of a CRT device and those patients receiving an upgrade of a permanent pacemaker or implantable defibrillator were included. In order to minimize the differences between patient characteristics, only those with CRTD are included in the analysis here (Quadripolar n = 357, Bipolar n = 364).

### Implant

CRT implantation was performed using a traditional approach in the cardiac catheter lab. The coronary sinus was cannulated using commercially available guiding sheaths and a suitable target coronary vein was identified with contrast venography. The LV leads were placed using an over‐the‐wire technique. The target vein was the posterolateral or lateral vein depending on venous anatomy, stability and stimulation thresholds with the avoidance of PNS. The decision to implant a bipolar or quadripolar lead was made by the implanting physician prior to the start of the procedure. In the case of bipolar LV leads, the subtype was arbitrarily assigned by the cardiac physiologist depending on local availability. Bipolar leads from all 3 major manufacturers (Boston Scientific Ltd., Medtronic Ltd., and St. Jude Medical Ltd.) were used, which have at least 4 different pacing configurations. The quadripolar lead used was the 1458Q, Quartet (St. Jude Medical), a preshaped 4.7F, over the wire, steroid eluting lead with 4 5.1F titanium nitride‐coated platinum–iridium alloy electrodes. The 3 ring electrodes are located 20, 30, and 47 mm from the distal tip electrode. When connected to the corresponding CRT defibrillator generator (Quadra Assura, Promote Q, Promote Quadra or Unify Quadra, St. Jude Medical) up to 10 different bipolar pacing configurations are available (6 bipolar and 4 extended bipolar with the right ventricular lead coil as the anode). The recent addition of a CRTP device (Allure Quadra RF PM3243, St. Jude Medical) compatible with the Quartet lead accounted for 32 cases. Furthermore, the recent licensing of the Attain Performa lead (Medtronic Ltd.) and associated devices (Brava Quad and Viva Quad, Medtronic Ltd.) accounted for 15 quadripolar cases. All procedures were performed under local anaesthesia with intravenous conscious sedation.

### PNS Testing

LV capture thresholds were tested in at least 1 vector at 0.5 milliseconds pulse width both during initial positioning of the lead and also via the generator following implantation. PNS was looked for by pacing at 10 volts (V) at pulse width 0.5 milliseconds in at least 1 vector. The final programmed pacing vector was set at the discretion of the implanting physician once satisfactory pacing parameters were achieved in addition to the absence of PNS. If PNS was present, a different vector was chosen with the same lead position; however, if there was no satisfactory vector the lead was repositioned within the same vein or moved to an alternate vein. The inability to deliver an LV lead into any venous branch without a reasonable threshold and avoidance of diaphragmatic pacing was defined as a failed implant.

Where there was intraprocedural difficulty with implantation due to coronary venous anatomy, high thresholds or PNS, the implanting physician had the option to change to a different lead. This may have been a change of lead from within the same manufacturer or between manufacturers. Data from Guy's and St Thomas’ revealed 40 cases (6%) where the first implanted LV lead was initially unsuccessful. For the second LV lead attempt, a different bipolar lead from the same manufacturer was used (n = 12), a bipolar lead from a different manufacturer was used (n = 8), a bipolar lead was swapped for a quadripolar lead (n = 9), or a quadripolar lead was swapped for a bipolar lead (n = 11). This represents a crossover rate of 1.4% from bipolar to quadripolar and 1.7% from quadripolar to bipolar leads.

All patients received a postprocedural chest x‐ray and a full check of device pacing thresholds, sensing and detection of PNS as well as a wound check. Thereafter, patients attended for a device check at 3 months, 6 months and then at a time interval determined by the cardiac physiologist in outpatient clinic depending on further pacing issues. PNS at follow‐up was defined as a regular sensation of diaphragmatic contraction reported by the patient and confirmed at follow‐up either at rest or following maneuvers at any pacing output. Where this could not be programmed around or if the lead threshold became unacceptably high in any configuration, patients were put forward for a lead revision. Transthoracic echocardiography was used to guide optimization of AV and VV delays in all patients implanted at Guy's and St Thomas's Hospital and at the discretion of the implanter or cardiologist following up the patient at the other centers. Mortality data were obtained through the respective hospital's local database. This is kept up to date via information from the NHS Trust, the patient's primary care physicians, and includes information from ambulatory care centers and other hospitals. We analyzed patients implanted between January 2009 and January 2014 allowing at least 6 months of follow‐up data per patient recorded.

### Data Analysis

All analyses were performed on PASW Statistics 21 (SPSS Inc, Chicago, IL, USA). Continuous variables with a Gaussian distribution were described using mean and standard error of the mean (SEM). Categorical data were described by an absolute number of occurrences and associated frequency (%). All continuous data passed a normality test and differences between these groups were determined using a two‐tailed, unpaired *t*‐test. Comparisons between categorical data were expressed as odds ratios and 95% confidence interval limits (CIL) and univariate analysis performed on binomial data using the χ^2^ test. Fisher's exact test was performed in cases of small group numbers. Data were dichotomized based on whether the implanting LV lead was quadripolar or bipolar. Kaplan–Meier analysis was performed to demonstrate survival time. A Cox logistical regression model was performed to analyze this effect of mortality over time. Furthermore, a multivariate analysis was performed to correct for demographic variables that were significantly different amongst bipolar and quadripolar groups. Results were considered significant at P < 0.05.

## Results

Seven hundred and twenty‐one patients (357 quadripolar, 364 bipolar) were implanted with a CRTD between January 2009 and January 2014 as shown in Table 1. Mean age was 68.4 ± 0.55 years in the quadripolar group and 69.8 ± 0.59 years in the bipolar group (P = 0.08). A total of 15.4% (n = 55) patients were female in the quadripolar group compared with 17.4% (n = 64) in the bipolar group (P = 0.43). An ischemic etiology of heart failure was found in a lower proportion of patients implanted with a quadripolar lead (54.1% vs. 62.6%, P = 0.02). Furthermore, sinus rhythm was more prevalent in those implanted with a quadripolar lead (95% vs. 86.8%, P < 0.001). Both groups had a similar percentage of biventricular pacing throughout the follow‐up period (94.7% ± 0.81% vs. 94.2% ± 1.04% quadripolar vs. bipolar group, P = 0.89).

**Table 1:** Demographic and Implantation Data and Mortality for CRTD Cases (n = 721)

	Quadripolar		Bipolar Lead		*t*‐Test or **χ^2^**
	(n = 357)		(n = 364)		P Value
Patients				
Age	68.4 ± 0.55		69.8 ± 0.59		0.08
Female gender	55	15.4%	64	17.4%	0.43
Ischemic heart disease	193	54.1%	228	62.6%	0.02
Sinus rhythm	339	95.0%	316	86.8%	<0.001
Mobitz II/Complete heart block	9	2.5%	14	3.8%	0.31
% Biventricular pacing	94.7 ± 0.81		94.2 ± 1.04		0.89
Procedure					
LV lead upgrade	8	2.2%	69	18.9%	<0.001
LV threshold (V)	1.27 ± 0.07		1.37 ± 0.04		0.03
LV pulse width (milliseconds)	0.50 ± 0.03		0.53 ± 0.05		0.09
LV lead impedance at implant (Ω)	850 ± 31		920 ± 29		<0.001
LV threshold at implant (μJ)	0.95 ± 0.04		1.08 ± 0.02		0.003
Fluoro dose (cGy cm^2^)	1,028 ± 59		1,950 ± 235		<0.001

Continuous data are presented mean ± standard error of the mean.

### Implantation Parameters

Upgrades to CRT systems in patients with existing devices accounted for 2.2% (n = 8) in the quadripolar group and 18.9% (n = 69) in the bipolar group (P < 0.001). LV lead final threshold was significantly lower in the quadripolar group (1.27 ± 0.07 V vs. 1.37 ± 0.04 V, P = 0.03) as was lead impedance (850 ± 31 ohms vs. 920 ± 29 ohms, P < 0.001). The mean pacing energy delivered was also calculated in microJoules (μJ) in order to take into account the varying pulse width durations used and lead impedances. Energy required to capture the LV lead was also significantly lower in the quadripolar compared with the bipolar group (0.95 ± 0.04 μJ vs. 1.08 ± 0.02 μJ, P = 0.003). The total radiation dose during implantation was 1,028 ± 59 cGy cm^2^ in the group implanted with quadripolar leads, far lower than those receiving bipolar leads, 1,950 ± 235 cGy cm^2^ (P < 0.001, Table 1).

### Complications

**Figure 1:**
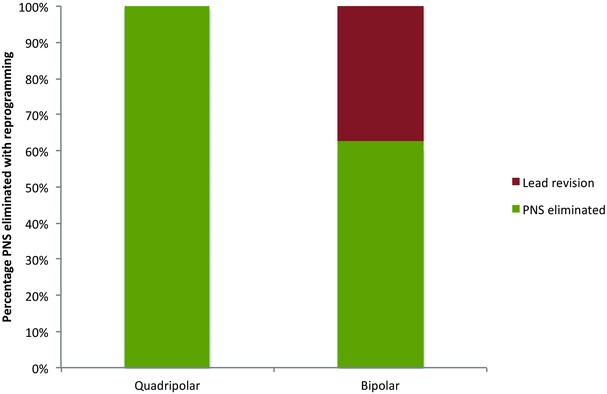
This chart demonstrates the outcomes for the cases of PNS between the 2 groups. A total of 100% of cases were successfully reprogrammed around in the quadripolar group compared with 60% (24/40) in the bipolar group. The remainder required LV lead revision.

Successful implantation of an LV lead was high and not significantly different between those receiving quadripolar and bipolar leads (96.1% vs. 95.1%, P = 0.51). PNS during the entire follow‐up was recorded in 55 (16.0%) patients with a quadripolar lead and 40 (11.6%) with a bipolar lead (P = 0.08) as shown in Table 2. In the quadripolar group, reprogramming eliminated all PNS; by comparison, only 24/40 (60%) of those with a bipolar lead were successfully reprogrammed and the remaining 40% required LV lead repositioning (see Fig. 1) (P < 0.001).

**Table 2:** Complications for CRTD Cases (n = 721)

	Quadripolar Lead		Bipolar Lead		Odds Ratio	*t*‐Test or **χ^2^**
	(n = 357)		(n = 364)		(95% CI)	P Value
Complications						
Successful LV lead implantation	343	96.1%	346	95.1%	1.28 (0.62–2.6)	0.51
Phrenic nerve stimulation (post implant)	55	16.0%	40	11.6%	1.48 (0.95–2.29)	0.08
PNS programmed around (post implant)	55	100%	24	60.0%	3.29 (2.36–4.60)	<0.001
LV lead displacement	6	1.7%	16	4.6%	0.37 (0.14–0.96)	0.03
LV lead repositioning	7	2.0%	18	5.2%	0.38 (0.16–0.93)	0.03
Wound infection (minor)	18	5.0%	30	8.7%	0.59 (0.32–1.08)	0.09
Wound infection (requiring intervention)	4	1.1%	8	2.0%	0.50 (0.15–1.70)	0.26
All‐cause mortality CRTD	47	13.2%	82	22.5%	0.64 (0.44–0.93)	<0.001
Mean follow‐up for CRTDs (days)	868 ± 22		890 ± 21			0.54

LV lead displacement was significantly less frequent among those implanted with a quadripolar lead (1.7% vs. 4.6%, P = 0.03), as was the proportion of LV lead repositioning required (2.0% vs. 5.2%, P = 0.03). Wound infection was defined by symptoms and signs of clinical infection and/or raised inflammatory markers with no other identifiable cause. This occurred at a similar frequency in both the quadripolar (5.0%, n = 18) and bipolar group (8.7%, n = 30, P = 0.09). Most of these episodes were successfully treated with targeted antimicrobial therapy; a similar frequency required reintervention in each group (1.1%/n = 4 vs. 2.0%/n = 8, quadripolar vs. bipolar, P = 0.26.) Table 2 summarizes these findings.

### Follow‐Up and Mortality

**Figure 2:**
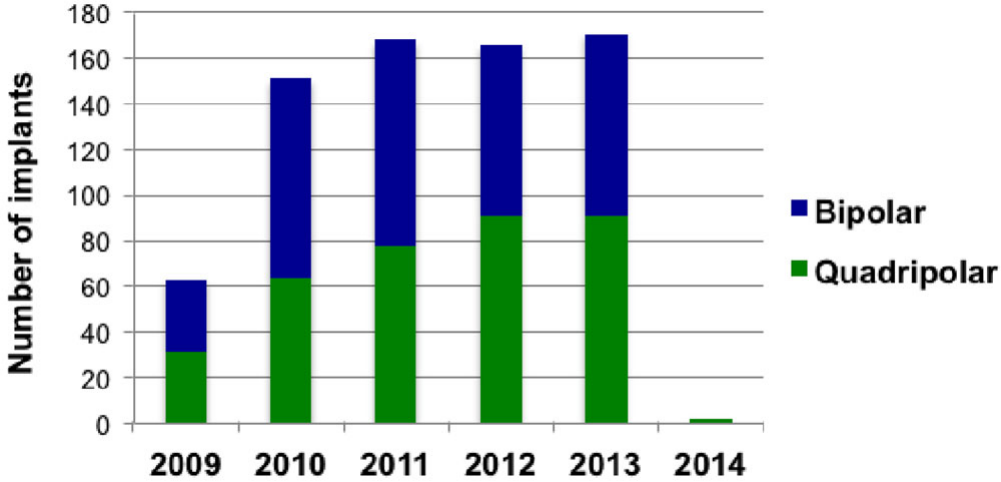
This bar chart demonstrates the year‐on‐year split of quadripolar versus bipolar CRTD devices implanted across the 3 UK centers. The first quadripolar leads were available in the latter third of 2009; data collection started at this point and therefore the total number of devices inserted was lower. The yearly insertion rate was similar across the other 4 years of study with a small increase in the uptake and use of quadripolar leads.

**Figure 3:**
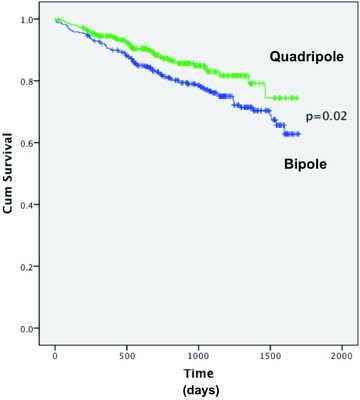
Kaplan–Meier survival curves of patients implanted with a CRTD. Quadripolar leads are denoted by the green line and bipolar leads by the blue line. Time from implant on the x‐axis (days).

Patients were followed up for at least 6 months; the last patient was implanted in January 2014 with an analysis date of 1 August 2014. The mean follow‐up period for all patients was 879 ± 14 days and was not significantly different between the 2 groups (Table 2). Figure 2 shows the yearly implantation figures for CRTD devices dichotomized by LV lead type. The quadripolar leads became available for implantation in the latter third of 2009 and therefore data collection began at this point; this explains the lower overall implantation figures for this year. Of the 721 CRTD devices implanted, overall mortality rate was significantly lower at 13.2% in the quadripolar group compared with 22.5% in the bipolar group (P < 0.001). When a Cox multivariate regression analysis was performed, quadripolar leads were associated with a lower hazard ratio (HR) of mortality compared with bipolar leads (HR 0.66, 95% CI: 0.46–0.95, P = 0.03). When correcting for the variables “ischemic heart disease” and “sinus rhythm,” which were significantly different between the groups on univariate analysis, there still remained a significant reduction in mortality associated with quadripolar leads on a multivariate analysis (HR 0.65, 95% CI: 0.46–0.96, P = 0.03). Figure 3 shows a Kaplan–Meier survival analysis demonstrating a persistently higher cumulative survival since CRT implantation in those implanted with a quadripolar lead (P = 0.02).

## Discussion

The main findings of this study are the following:

1. In those patients with PNS, this was entirely eliminated by re‐programming in the quadripolar group, while 40% of those from the bipolar lead group required LV lead revision in order to achieve successful long‐term CRT delivery.
2. LV lead displacement and repositioning was significantly less frequent amongst those patients implanted with a quadripolar lead.
3. Quadripolar LV leads had a lower implantation threshold and pacing energy compared with conventional bipolar leads.
4. The mean radiation dose for the implant procedures when quadripolar leads were used was around half of that received by patients when bipolar leads were used.
5. Overall all‐cause mortality among the CRT‐D cohort was significantly lower in the quadripolar cohort as compared with the bipolar group.

Despite advances in technology and improving clinical expertise, the placement of an LV lead remains technically difficult. This is often due to variable coronary venous anatomy and the ability to achieve good pacing thresholds without PNS. The flexibility of having different LV pacing configurations is a helpful feature of CRT devices in preventing high thresholds and PNS.^12^ Having an LV lead with multiple pacing electrodes gives the implanting physician the best chance of achieving this. Initial experience in small, short‐term studies with quadripolar leads report a high frequency of procedural success and low rates of displacement, procedural complications and PNS.^10^, ^11^, ^13^, ^14^ However, many complications and reinterventions occur at a significant period following the immediate implantation. Here we present the findings of a large multicenter registry of CRT patients directly comparing quadripolar to bipolar leads, with follow‐up averaging 29 months (minimum of 6 months).

First, our data demonstrate similar implantation lead parameters to other groups^10^ with significantly lower thresholds and impedance values as compared with bipolar leads. Furthermore, the energy (in microJoules) required to capture the LV lead was also significantly lower with the quadripolar leads and this is likely to lead to increased device longevity.^11^

Second, radiation doses (reported here as the dose area product, measured in cGy cm^2^) were significantly lower in the quadripolar group as compared with the bipolar group. While total procedural time was not accurately recorded, documentation of radiation dose is a legal requirement and as such is accurately recorded in all UK centers. It is likely that greater programmability of the quadripolar lead contributed to a lower screening time and thus radiation dose.

The frequency of PNS over the follow‐up period was surprisingly slightly higher among the quadripolar cohort, although this did not reach statistical significance. This might be related to physicians feeling that they have greater freedom to place leads more distally in the most suitable vein in order to achieve better stability with the knowledge that more proximal pacing vectors are available if necessary. However, when looking at the ability to reprogram around PNS, there was complete elimination of this in the quadripolar group. In the bipolar cohort on the other hand, PNS persisted in 40% of cases despite reprogramming and subsequently required lead revision. These data are consistent with similar reports of elimination of PNS with reprogramming of the Quartet lead. The rates of lead displacement and repositioning were higher in the bipolar group; again these data are concordant with prior studies showing similar trends. The rates of wound infection were similar amongst groups and a small, similar proportion required reintervention.

Finally, we calculated the mortality of the CRTD cohort (n = 721). We chose to only analyze those with a defibrillator *in situ* in order to minimize differences in patient characteristics. All CRTD patients met criteria for implantation based on current guidelines.^15^ We demonstrated that all‐cause mortality was significantly lower among patients implanted with a quadripolar LV lead (13.2%) compared with a bipolar lead (22.5%) over a similar follow‐up period. While the age and sex distribution of this subgroup were similar and both groups had a similar percentage of biventricular pacing at follow‐up, there were a higher proportion of patients with an ischemic etiology in the bipolar group. We do not have data on the volume of myocardial scar in each group, which can strongly influence clinical outcome.^16^ The proportion of patients with sinus rhythm was also lower in the bipolar cohort, suggesting this group had a higher proportion of atrial arrhythmia. As such, having atrial fibrillation may have put this group at higher risk of poorer outcomes.^17^ Despite the potential confounders, there remains a substantial difference in absolute mortality between those patients implanted with a quadripolar lead and those with a bipolar lead (9.3% absolute and 41% relative difference in mortality), even when correcting for ischemic etiology and sinus rhythm in a multivariate analysis.

## Limitations

This large cohort of 721 patients is registry data, not randomized and therefore prone to the effects of confounding variables. Unfortunately, we do not have comprehensive data on NYHA class, QRS duration/morphology, or left ventricular ejection fraction, which was often obtained at referring centers. All patients fulfilled conventional CRTD implantation criteria and therefore were NYHA II/III with severely impaired LV systolic function (or moderately impaired if there was Mobitz II or complete heart block, which was not different between the groups). In those patients where data was readily available (n = 439), no difference in mean electronically measured QRS duration (Quadripolar 162 ± 1.6 milliseconds vs. Bipolar 159 ± 1.86 milliseconds, P = 0.23), or patients with NYHA class III symptoms (Quadripolar 140 [78%] vs. Bipolar 210 [81%], P = 0.40) was observed. These data represent real world clinical practice in the United Kingdom and provide insight into the associated differences between the quadripolar and bipolar groups. A randomized study will help to answer the clinical question of which of the 2 lead types is more effective. The MORE‐CRT trial (clinicaltrials.gov number NCT01510652) is comparing the clinical outcomes during follow‐up, of quadripolar and bipolar LV pacing, and is ongoing.

## Conclusions

In a large multicenter cohort of heart failure patients implanted with CRT, the use of a quadripolar LV lead is associated with elimination of PNS compared with conventional bipolar LV leads. Furthermore, there is a lower mortality associated with use of a quadripolar lead compared with bipolar leads in conjunction with a defibrillator system. Further randomized studies are needed to assess whether implantation using quadripolar leads is superior and should become the standard of care.

## References

[1] Berry C , Doughty RN , Granger C , Køber L , Massie B , McAlister F , McMurray J , Pocock S , Poppe K , Swedberg K , Somaratne J , Whalley GA , Ahmed A , Andersson B , Bayes‐Genis A , Berry C , Cowie M , Cubbon R , Doughty RN , Ezekowitz J , Gonzalez‐Juanatey J , Gorini M , Gotsman I , Grigorian‐Shamagian L , Guazzi M , Kearney M , Køber L , Komajda M , di Lenarda A , Lenzen M , Lucci D , Macín S , Madsen B , Maggioni A , Martínez‐Sellés M , McAlister F , Oliva F , Poppe K , Rich M , Richards M , Senni M , Squire I , Taffet G , Tarantini L , Tribouilloy C , Troughton R , Tsutsui H , Whalley GA , Doughty RN , Earle N , Perera K , Poppe K , Whalley GA , Dobson J , Pocock S , Poppe K , Doughty RN , Whalley G , Andersson B , Hall C , Richards AM , Troughton R , Lainchbury J , Berry C , Hogg K , Norrie J , Stevenson K , Brett M , McMurray J , Pfeffer MA , Swedberg K , Granger CB , Held P , McMurray JJ , Michelson EL , Olofsson B , Östergren J , Yusuf S , Køber L , Torp‐Pedersen C , Ahmed A , Lenzen MJ , Scholte OP , Reimer WJ , Boersma E , Vantrimpont PJ , Follath F , Swedberg K , Cleland J , Komajda M , Gotsman I , Zwas D , Planer D , Azaz‐Livshits T , Admon D , Lotan C , Keren A , Grigorian‐Shamagian L , Varela‐Roman A , Mazón‐Ramos P , Rigeiro‐Veloso P , Bandin‐Dieguez MA , Gonzalez‐Juanatey JR , Guazzi M , Myers J , Arena R , McAlister FA , Ezekowitz J , Armstrong PW , Cujec B , Paterson I , Cowie MR , Wood DA , Coats AJ , Thompson SG , Suresh V , Poole‐Wilson PA , Sutton GC , Martínez‐Sellés M , Robles JA , Prieto L , Muñoa MD , Frades E , Díaz‐Castro O , Almendral J , Tarantini L , Faggiano P , Senni M , Lucci D , Bertoli D , Porcu M , Opasich C , Tavazzi L , Maggioni AP , Kirk V , Bay M , Parner J , Krogsgaard K , Herzog TM , Boesgaard S , Hassager C , Nielsen OW , Aldershvile J , Nielsen H , Kober L , Macín SM , Perna ER , Canella JP , Alvarenga P , Pantich R , Ríos N , Farias EF , Badaracco JR , Madsen BK , Hansen JF , Stokholm KH , Brons J , Husum D , Mortensen LS , Bayes‐Genis A , Vazquez R , Puig T , Fernandez‐Palomeque C , Bardají A , Pascual‐Figal D , Ordoñez‐Llanos J , Valdes M , Gabarrus A , Pavon R , Pastor L , Gonzalez‐Juanatey JR , Almendral J , Fiol M , Nieto V , Macaya C , Cinca J , Bayes de Luna A , Newton JD , Blackledge HM , Squire IB , Wright SP , Whalley GA , Doughty RN , Kerzner R , Gage BF , Freedland KE , Rich MW , Huynh BC , Rovner A , Freedland KE , Carney RM , Rich MW , Taffet GE , Teasdale TA , Bleyer AJ , Kutka NJ , Luchi RJ , Tribouilloy C , Rusinaru D , Mahjoub H , Soulière V , Lévy F , Peltier M , Tsutsui H , Tsuchihashi M , Takeshita A , MacCarthy PA , Kearney MT , Cubbon R , Nolan J , Lee AJ , Prescott RJ , Shah AM , Brooksby WP , Fox KA , Varela‐Roman A , Gonzalez‐Juanatey JR , Basante P , Trillo R , Garcia‐Seara J , Martinez‐Sande JL , Gude F : Meta‐analysis Global Group in Chronic Heart Failure (MAGGIC) . The survival of patients with heart failure with preserved or reduced left ventricular ejection fraction: An individual patient data meta‐analysis . Euro Heart J 2012;33:1750‐1757. 10.1093/eurheartj/ehr254 21821849

[2] Cleland JGF , Daubert J‐C , Erdmann E , Freemantle N , Gras D , Kappenberger L , Tavazzi L : Cardiac Resynchronization‐Heart Failure (CARE‐HF) Study Investigators. The effect of cardiac resynchronization on morbidity and mortality in heart failure. N Engl J Med 2005;352:1539‐1549. 15753115 10.1056/NEJMoa050496

[3] Abraham WT , Fisher WG , Smith AL , Delurgio DB , Leon AR , Loh E , Kocovic DZ , Packer M , Clavell AL , Hayes DL , Ellestad M , Trupp RJ , Underwood J , Pickering F , Truex C , McAtee P , Messenger J : MIRACLE Study Group. Multicenter InSync Randomized Clinical Evaluation. Cardiac resynchronization in chronic heart failure. N Engl J Med 2002;346:1845‐1853. 12063368 10.1056/NEJMoa013168

[4] Biffi M , Exner DV , Crossley GH , Ramza B , Coutu B , Tomassoni G , Kranig W , Li S , Kristiansen N , Voss F : Occurrence of phrenic nerve stimulation in cardiac resynchronization therapy patients: The role of left ventricular lead type and placement site. Europace 2013;15:77‐82. 22848075 10.1093/europace/eus237

[5] Shetty AK , Duckett SG , Bostock J , Roy D , Ginks M , Hamid S , Rosenthal E , Razavi R , Rinaldi CA : Initial single‐center experience of a quadripolar pacing lead for cardiac resynchronization therapy. Pacing Clin Electrophysiol 2011;34:484‐489. 21208241 10.1111/j.1540-8159.2010.03003.x

[6] Biffi M , Foerster L , Eastman W , Eggen M , Grenz NA , Sommer J , De Santo T , Haddad T , Varbaro A , Yang Z : Effect of bipolar electrode spacing on phrenic nerve stimulation and left ventricular pacing thresholds: An acute canine study. Circulation 2012;5:815‐820. 22787012 10.1161/CIRCEP.112.971317

[7] Attain Performa product information, Medtronic . 2013 March 29; pp. 1‐2.

[8] Biffi M , Moschini C , Bertini M , Saporito D , Ziacchi M , Diemberger I , Valzania C , Domenichini G , Cervi E , Martignani C , Sangiorgi D , Branzi A , Boriani G : Phrenic stimulation: A challenge for cardiac resynchronization therapy. Circulation 2009;2:402‐410. 19808496 10.1161/CIRCEP.108.836254

[9] Shetty AK , Mehta P , Bostock J , Rinaldi CA : Quad‐site pacing using a quadripolar left ventricular pacing lead. Pacing Clin Electrophysiol 2013;36:e48‐e50. 22126629 10.1111/j.1540-8159.2011.03267.x

[10] Forleo GB , Mantica M , Di Biase L , Panattoni G , Rocca Della DG , Papavasileiou LP , Santamaria M , Santangeli P , Avella A , Sergi D , Santini L , Tondo C , Natale A , Romeo F : Clinical and procedural outcome of patients implanted with a quadripolar left ventricular lead: Early results of a prospective multicenter study. Heart Rhythm 2012;9:1822‐1828. 22841876 10.1016/j.hrthm.2012.07.021

[11] Sperzel J , Dänschel W , Gutleben K‐J , Kranig W , Mortensen P , Connelly D , Trappe H‐J , Seidl K , Duray G , Pieske B , Stockinger J , Boriani G , Jung W , Schilling R , Saberi L , Hallier B , Simon M , Rinaldi CA : First prospective, multi‐centre clinical experience with a novel left ventricular quadripolar lead. Europace 2012;14:365‐372. 21993431 10.1093/europace/eur322

[12] Seifert M , Schau T , Moeller V , Neuss M , Meyhoefer J , Butter C : Influence of pacing configurations, body mass index, and position of coronary sinus lead on frequency of phrenic nerve stimulation and pacing thresholds under cardiac resynchronization therapy. Europace 2010;12:961‐967. 20444725 10.1093/europace/euq119

[13] Tomassoni G , Baker J , Corbisiero R , Love C , Martin D , Niazi I , Sheppard R , Worley S , Beau S , Greer GS , Aryana A , Cao M , Harbert N , Zhang S , for the Promote® Q CRT‐D and Quartet® Left Ventricular Heart Lead Study Group : Postoperative performance of the Quartet ®Left ventricular heart lead. J Cardiovasc Electrophysiol 2013;24:449‐456. 23339555 10.1111/jce.12065

[14] Mehta PA , Shetty AK , Squirrel M , Bostock J , Rinaldi CA : Elimination of phrenic nerve stimulation occurring during CRT: Follow‐up in patients implanted with a novel quadripolar pacing lead. J Interv Card Electrophysiol 2012;33:43‐49. 21833513 10.1007/s10840-011-9598-5

[15] Authors/Task Force Members , Brignole M , Auricchio A , Baron‐Esquivias G , Bordachar P , Boriani G , Breithardt O‐A , Cleland J , Deharo JC , Delgado V , Elliott PM , Gorenek B , Israel CW , Leclercq C , Linde C , Mont L , Padeletti L , Sutton R , Vardas PE , ESC Committee for Practice Guidelines (CPG) , Zamorano JL , Achenbach S , Baumgartner H , Bax JJ , Bueno H , Dean V , Deaton C , Erol C , Fagard R , Ferrari R , Hasdai D , Hoes AW , Kirchhof P , Knuuti J , Kolh P , Lancellotti P , Linhart A , Nihoyannopoulos P , Piepoli MF , Ponikowski P , Sirnes PA , Tamargo JL , Tendera M , Torbicki A , Wijns W , Windecker S , Document Reviewers , Kirchhof P , Blomstrom Lundqvist C , Badano LP , Aliyev F , Bansch D , Baumgartner H , Bsata W , Buser P , Charron P , Daubert JC , Dobreanu D , Faerestrand S , Hasdai D , Hoes AW , Le Heuzey JY , Mavrakis H , McDonagh T , Merino JL , Nawar MM , Nielsen JC , Pieske B , Poposka L , Ruschitzka F , Tendera M , Van Gelder IC , Wilson CM : 2013 ESC Guidelines on cardiac pacing and cardiac resynchronization therapy: The Task Force on cardiac pacing and resynchronization therapy of the European Society of Cardiology (ESC). Developed in collaboration with the European Heart Rhythm Association (EHRA). Euro Heart J 2013;34:2281‐2329. 10.1093/eurheartj/eht150 23801822

[16] Ypenburg C , Roes SD , Bleeker GB , Kaandorp TAM , de Roos A , Schalij MJ , van der Wall EE , Bax JJ : Effect of total scar burden on contrast‐enhanced magnetic resonance imaging on response to cardiac resynchronization therapy. AJC 2007;99:657‐660. 10.1016/j.amjcard.2006.09.115 17317367

[17] Rivero‐Ayerza M , Scholte Op Reimer W , Lenzen M , Theuns DA , Jordaens L , Komajda M , Follath F , Swedberg K , Cleland JG : New‐onset atrial fibrillation is an independent predictor of in‐hospital mortality in hospitalized heart failure patients: Results of the EuroHeart Failure Survey. Euro Heart J 2008;29:1618‐1624. 10.1093/eurheartj/ehn217 18515809

